# Volumetric Computed Tomography Angiography in the Evaluation of Mediastinal Fluid Collections following Congenital Cardiac Surgery

**DOI:** 10.1155/2013/426923

**Published:** 2013-01-28

**Authors:** Arno A. W. Roest, Joost Roelofs, Mark G. Hazekamp, Marry E. B. Rijlaarsdam, Jacob Geleijns, Lucia J. M. Kroft

**Affiliations:** ^1^Department of Pediatric Cardiology, Leiden University Medical Center, Albinusdreef 2, P.O. Box 9600, 2300 RC Leiden, The Netherlands; ^2^Department of Radiology, Leiden University Medical Center, Albinusdreef 2, P.O. Box 9600, 2300 RC Leiden, The Netherlands; ^3^Department of Cardiothoracic Surgery, Leiden University Medical Center, Albinusdreef 2, P.O. Box 9600, 2300 RC Leiden, The Netherlands

## Abstract

We present 3 patients with 4 causes of mediastinal fluid collection after congenital cardiac surgery in this extended case report. Volumetric computed tomography played an essential role in diagnosing causes and extent, relevant to subsequent management. Recent advances in volumetric computed tomography allow fast and accurate imaging of cardiovascular and extravascular structures in children with acceptable radiation dose, providing a powerful imaging tool for the evaluation of complications after congenital cardiac surgery.

## 1. Introduction

Congenital heart disease (CHD) is the most frequent congenital abnormality with an estimated incidence of moderate-to-severe CHD of 6 per 1000 live births [1]. A substantial part of patients with moderate-to-severe CHD require surgical intervention, which is currently performed with low postoperative mortality [2]. Mediastinal fluid collection is an infrequent but potentially lethal complication after congenital cardiac surgery, and correct identification of the cause is essential with important implications for management. Echocardiography is the first-line imaging tool in the postoperative followup of patients with CHD [2]. However, the use of echocardiography is hampered by operator dependency, variable acoustic window, small field of view, and the inability to penetrate air and bones [3–5]. Recently, volumetric computed tomography (CT) angiography has emerged as a valuable tool to assess complications after congenital cardiac surgery [4, 6, 7]. Volumetric CT is performed with a CT scanner with a detector that covers a large volume with a field of view up to 50 cm diameter and a length of up to 16 cm.

In this case report, we present 3 patients with mediastinal fluid collections following congenital cardiac surgery. We demonstrate the value of volumetric CT in the identification of the origin and extent of these collections helping decision making for subsequent patient management.

## 2. Case  1

**Figure 1:**
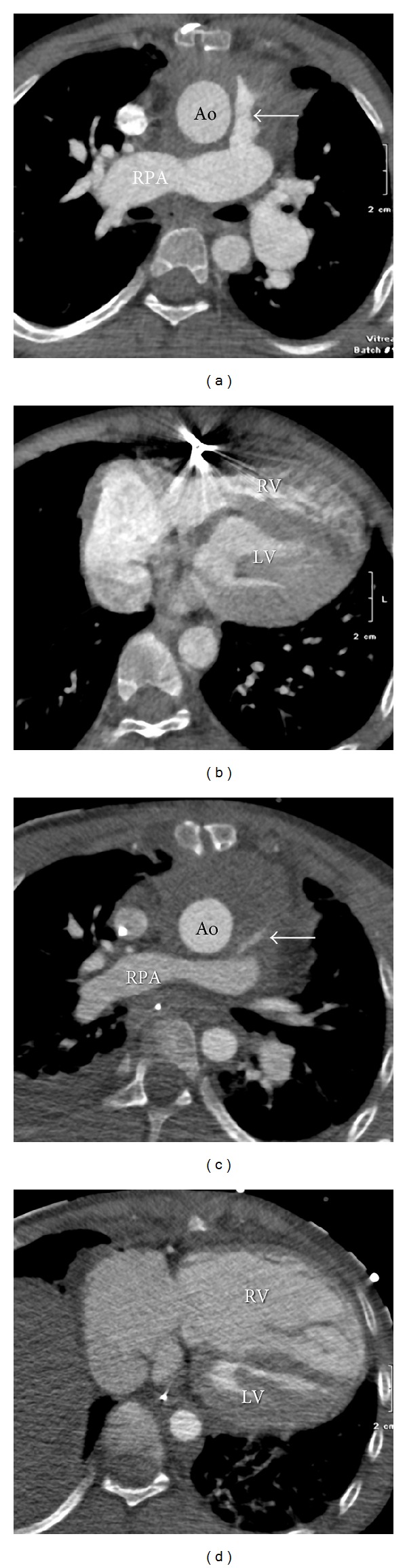
Mediastinitis after Contegra conduit and late complication of large mediastinal fluid collection. ECG-triggered volumetric contrast-enhanced cardiac CT (AquilionONE, Toshiba medical systems, Otawara, Japan) is acquired at end systole (45% of cardiac RR cycle). Transverse views in 8-year male patient with moderate soft tissue/fluid collection around ascending aorta (Ao, in (a)). Relative stenosis of Contegra conduit (arrow, (a)). Patient also had osteolytic destruction of parts of sternum compatible with osteomyelitis (not shown). Diagnosis mediastinitis was based on CT findings and clinical findings. Ventricles at lower level (b). Nine months later; large mediastinal fluid collection and subtotal compression on the Contegra conduit (arrow, (c)). Note the secondary massive right ventricle (RV) dilatation due to outflow obstruction (d). Ao: aorta; LV: left ventricle; RPA: right pulmonary artery. Dose-length products of the CT scans were 26.4 mGy*·*cm for the first scan (a, b) and 29.5 mGy*·*cm for the second scan (c, d). Correction factor for chest CT at 100 kV for 8 years age was 0.026 mSv·mGy^−1^·cm^−1^; effective doses *E* were 0.7 mSv and 0.8 mSv, respectively.

**Figure 2:**
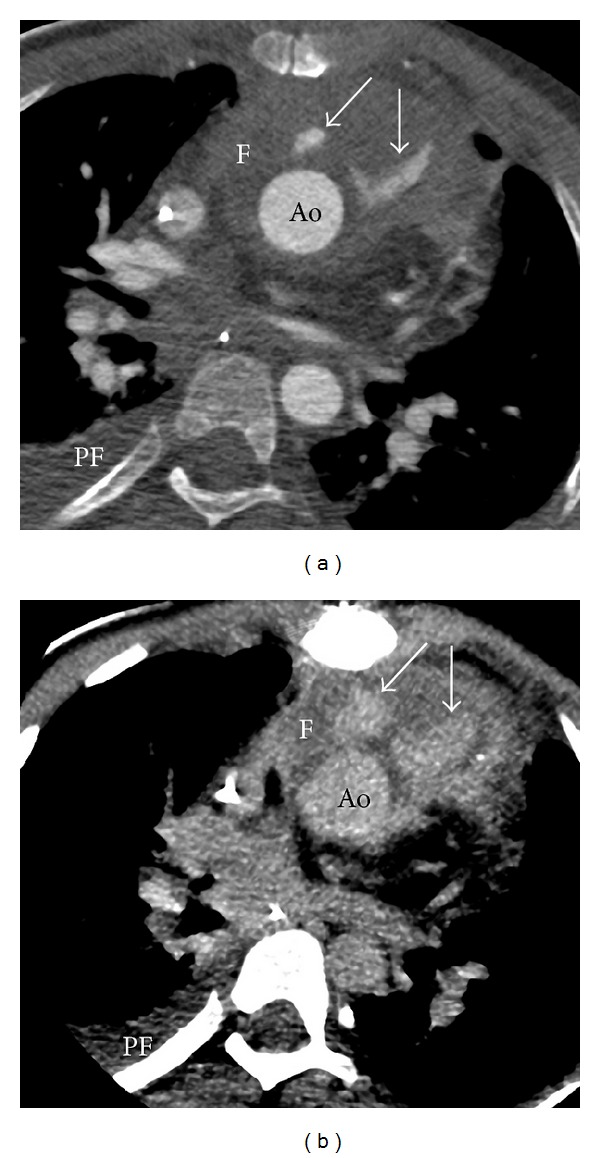
Active blood extravasation due to suture leakage. Same patient as in Figure 1 showing active extravasation of contrast (which is leakage of blood, arrows in (a) and (b)) ventral to the aorta (Ao) caused by suture leakage during chronic mediastinitis. Note accumulation of blood during delayed imaging (arrows, (b)). F: mediastinal fluid; PF: pleural fluid. Effective dose *E* = 0.8 mSv.

An 8 years and 2 months old male patient presented with increasing presternal swelling and mildly increased CRP of 31 mg/L. The patient had a history of truncus arteriosus (TA) with 22q11 deletion. The TA was corrected at the age of 2 weeks; the truncal valve was replaced by a bileaflet mechanical valve at the age of 1 year. At the age of 8 years, the right ventricular to pulmonary artery conduit was replaced by valve-containing Contegra conduit and, aortic valve replacement was done with a mechanical valve. Four months prior to admittance a pacemaker was placed because of complete atrioventricular block. The week before admittance, the parents noted a swelling at the sternotomy scar, which increased during the week, and was suspected for an infectious process or hematoma. Computed tomography (CT) imaging was performed for further evaluation. With CT, diagnosis of mediastinitis was made with moderate compression of Contegra conduit (Figures 1(a) and 1(b)). The next day the mediastinum was surgically explored, confirming extensive mediastinitis. Purulent effusion was removed, and omentum plasty was performed with application of gentamicin pearls. The patient was treated with intravenous antibiotics for an extensive period. Nine months later, the patient was admitted to the intensive care unit because of low blood pressure and seizures. CT imaging showed large amount of mediastinal fluid with severe narrowing of the Contegra conduit, and massive right ventricular dilatation due to outflow obstruction by subtotal conduit obstruction (Figures 1(c) and 1(d)). The mediastinal fluid collection was diagnosed as being active extravasation caused by suture leakage, most likely due to chronic mediastinitis (Figure 2). Surgical relieve was performed and sutures were placed on the proximal anastomosis of the vascular prosthesis. 

Currently, the patient is regularly seen at the outpatient clinic and has recovered well, although the preexisting neurological deficits in walking and speech had slightly worsened. 

## 3. Case  2

**Figure 3:**
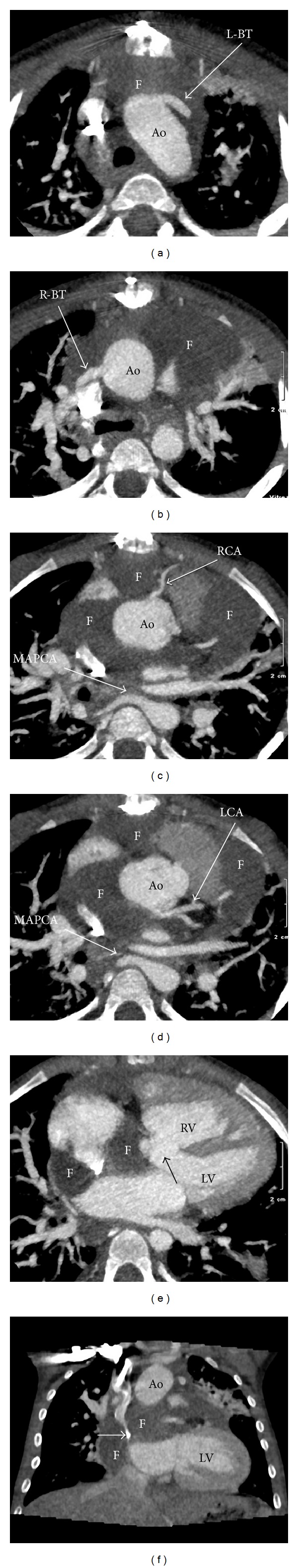
Seroma formation after modified Blalock-Taussig shunt. ECG-triggered volumetric contrast-enhanced cardiac CT (AquilionONE, Toshiba medical systems) is acquired in a single heart beat during systole at a heart rate of 127 beats per minute. Transverse orientation ((a)–(e)) and coronal orientation (f). 3 years old female patient after repair of pulmonary atresia by unifocalization and bilateral modified Blalock-Taussig shunt (BT). Extensive mediastinal fluid (F, all images) in the upper part of mediastinum. Note the excellent image quality showing the left BT shunt (L-BT, (a)) and right-BT shunt (R-BT, (b)), right coronary artery (RCA, (c)), left coronary artery (LCA, (d)), major aorta to pulmonary collateral arteries (MAPCA in c, (d)), and large subaortic ventricular septum defect (black arrow, (e)). The superior vena cava was severely compressed by the mediastinal fluid collection (white arrow, (f)). Ao: aorta; LV: left ventricle; RV: right ventricle. Dose-length product of the CT scan was 20.9 mGy*·*cm. Correction factor for chest CT at 100 kV for 3 years age was 0.039 mSv·mGy^−1^·cm^−1^; effective dose *E* was 0.8 mSv.

A three-year-old girl was admitted to the hospital because of suspected mediastinal fluid that was observed during routine echocardiography. The patient was known with pulmonary atresia with major aorta to pulmonary collateral arteries. Unifoclization with the use of a modified Blalock Taussig shunt on the left side was performed at the age of 1 month and on the right side at 7 months of age. Replacement of bilateral modified Blalock-Taussig shunts was done 1 month before admittance. CT imaging was performed to assess the nature and extent of the mediastinal fluid. Extensive mediastinal fluid collection, without any signs of mediastinitis, was found; it severely compressed the superior caval vein. All relevant anatomic structures and surgical shunts were evaluated (Figure 3). The mediastinal fluid collection was punctured and percutaneously drained. Analysis of the fluid revealed seroma, leading to the final diagnosis of perigraft seroma formation around the modified Blalock Taussig shunt.

Currently the patient is doing well and scheduled for correction with connection of the right ventricle to the pulmonary arterial system.

## 4. Case  3

**Figure 4:**
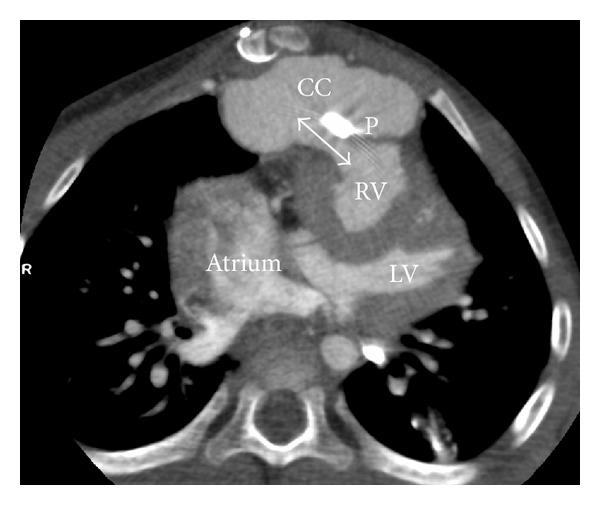
Pseudoaneurysm after surgical repair. ECG-triggered volumetric contrast-enhanced cardiac CT (AquilionONE, Toshiba medical systems) acquired during systole within a single heart beat at a heart rate of 109 beats per minute, in 2 years female patient. Image in transverse orientation. Large precardiac contrast collection (CC) with open connection (two-sided arrow) to the right ventricle (RV), at the site of RV patch (P). CT differential diagnosis was RV patch aneurysm or pseudoaneurysm. Surgery confirmed pseudoaneurysm. Dose-length product of the CT scan was 18.2 mGy·cm. Correction factor for chest CT at 100 kV for 2 years age was 0.044 mSv·mGy^−1^·cm^−1^; effective dose *E* was 1.4 mSv.

A 2-year-old girl was admitted because of mediastinal fluid observed during routine echocardiography. The girl was known with hypoplastic left heart syndrome after Norwood-procedure with placement of a Sanoconduit between the right ventricle and pulmonary arteries, followed by bidirectional cavopulmonary connection with closure of the Sanoshunt. To assess the nature and extent of the mediastinal fluid collection, CT imaging was performed. On CT, a large precardiac fluid collection with open connection to the right ventricle was found (Figure 4). CT differential diagnosis was right ventricular patch aneurysm or pseudoaneurysm. At surgery, pseudoaneurysm formation was confirmed at the site of previous anastomosis of the Sanoshunt to the right ventricle that had been closed by a patch. The pseudoaneurysm was removed. Currently, the patient is scheduled for completion of the Fontan circulation by connecting the inferior caval vein to the right pulmonary artery.

## 5. Discussion

Postoperative mediastinal fluid collections following congenital cardiac surgery may include pus in mediastinitis, seroma, aneurysm, or pseudoaneurysm or hemorrhage. 

Mediastinitis is probably the most common cause for mediastinal fluid collection after cardiac surgery, occurring in 1.4% following sternotomy in children [8]. Gram-positive organisms are most frequently found, although mediastinitis from Gram-negative organisms is increasingly recognized, especially in neonates, and is related to delayed sternal closure [8]. After diagnosing mediastinitis as the cause of mediastinal fluid collection, surgical debridement is performed in nearly all cases [8–10]. Knowledge on the cause and extent of mediastinal fluid is essential to guide clinical decision making. CT is very helpful in diagnosing mediastinitis [11, 12], especially from day 14 after sternotomy [13]. 

Several complications can occur after placement of a modified Blalock-Taussig shunt (mBTs), used in the palliation of cyanotic heart disease. Besides shunt thrombosis, perigraft seroma can develop, which is defined as a sterile collection of fluid in a nonsecretory wall surrounding a shunt [14]. The perigraft seroma can present as mediastinal fluid and can lead to airway compression and stenosis of the superior caval vein, as observed in case 2, due to the mass effect on adjacent mediastinal structures, and may even lead to cardiac tamponade [15]. The etiology is unclear and has been related to heparine use, pressure difference between the systemic and pulmonary arteries, and properties of the polytetrafluoroethylene material that is used in modified Blalock Tausig shunts [15, 16]. Mediastinal fluid due to perigraft seroma should be differentiated from mediastinitis as management is different. The seroma can be successfully drained percutaneously [15] with resolution of the symptoms as observed in case 2. Echocardiography is first-line imaging tool for the detection of perigraft seroma [14]. However, CT is helpful in distinguishing between seroma and abcess, in the evaluation of the extent of fluid collection and the effect on surrounding structures, and in planning the optimal location for drainage [17, 18].

After cardiac surgery formation of aneurysms is a rare but life-threatening complication [16]. The modified Norwood-Sano operation is one of the treatment options for the hypoplastic left heart syndrome and aneurysm formation near the proximal connection of the Sano-shunt to the right ventricle has been described [19, 20]. In our case, the use of CT angiography clearly depicted bright contrast in the mediastinal fluid collection, indicating aneurysm or pseudo-aneurysm with direct connection between the right ventricle and the contrast collection representing blood. This differentiates (pseudo) aneurysms from pus collections (abcess) and seroma. Knowledge on the location and extent of the aneurysm is essential for planning surgical intervention, because location of the aneurysm directly behind the sternum has important implications for the surgical approach [16]. 

Hemorrhage after cardiac surgery can also present as a mediastinal fluid collection [21]. Active mediastinal blood extravasation can be life threatening and thus requires fast and accurate diagnosis, as performed with CT in case 1. The CT observation of extravascular and extracardiac contrast depots that increase during delayed imaging (Figure 2) indicates active extravasation of blood that requires prompt surgical intervention.

CT has emerged as a valuable tool in the assessment of cardiac and vascular abnormalities in patients with congenital heart disease, especially in patients in whom echocardiography or cardiac magnetic resonance imaging is contraindicated or suboptimal, because of pacemaker-dependency or susceptibility to artifacts of devices such as coils or stents. In these cases, CT can be of great help. However, the use of radiation in CT is of concern, especially in children. The recent introduction of volumetric CT techniques that allow full cardiac or chest imaging in a single gantry rotation markedly reduces imaging time and radiation dose in children compared to conventional CT. In our cases presented, effective radiation doses varied between 0.7 and 1.4 mSv. Effective doses were calculated by using appropriate conversion factors adapted for age in these children [22]. Because of fast acquisition within 0.35 second and good temporal resolution of 175 milliseconds using a half reconstruction technique, the image quality is excellent. 

## 6. Conclusion

We present 3 cases with 4 causes of mediastinal fluid collection after congenital cardiac surgery, each with their specific CT findings. The importance of differentiating between the different causes of mediastinal fluid collection and the location and extent of the fluid is stressed by differences in management, ranging from percutaneous drainage of the fluid to immediate surgical intervention. Volumetric cardiac CT angiography is a fast and an accurate imaging tool with acceptable radiation dose for assessing complications after congenital cardiac surgery presenting as mediastinal fluid collection.
